# Measuring the performance of health systems in Latin America

**DOI:** 10.1093/eurpub/ckac131.308

**Published:** 2022-10-25

**Authors:** S Flores, M San Sebastian, P De Alva

**Affiliations:** 1 Institute for Public Health and Caring Science, Uppsala University, Uppsala, Sweden; 2 Department of Epidemiology and Global Health, Umeå University, Umeå, Sweden

## Abstract

**Background:**

Efficiency has been identified as a key intermediate policy objective for Universal Health Coverage. Despite that, it was estimated that 20 to 40% of health sector resource utilization is wasteful globally. An efficient use of existing resources in healthcare is critical and a priority policy in sustaining positive health outcomes for the population. This study aimed to perform a longitudinal efficiency analysis was found concerning health systems specific to all the Latin America region over the last 13 years.

**Methods:**

Health Adjusted Life Expectancy (HALE), Diphtheria Pertussis Tetanus (DPT)Immunization and Survivability Rate of under-5 were chosen as outputs, while Pooled Health Expenditure, Gross Domestic Product (GDP) per Capita and Population over 65 were selected as inputs. Cross sectional Data Envelopment Analysis using five-year averages and a longitudinal Data Envelopment Analysis (DEA) were performed creating several model iterations with our selected indicators. All the models had an output orientation, adjusted for variable returns to scale and incorporating a five-year time lag between inputs and outputs.

**Results:**

Our cross-sectional DEA Analysis found that the best performing country ended up being Nicaragua, followed by Cuba, Honduras, Costa Rica and Chile, while the countries performing the worst were Suriname, Venezuela and Guatemala. When observing efficiency scores behaviors longitudinally, all our model iterations result in the region decreasing efficiency in their health systems by 2.5% to 6.9% from 2000 to 2013, depending on the model chosen.

**Conclusions:**

The study reveals that all countries in Latin American can improve their health systems efficiency performance to different extents. Latin America reduced total health system productivity between 2000 and 2013. Further studies are required to uncover the extent and causes of this regression. A similar analysis in Europe may be warranted and could aid policy making.

**Key messages:**

• Overall efficiency in Latin American health systems has decreased by 3% to 7% over the last thirteen years.

• Nicaragua, Cuba, Honduras and Costa Rica seem to have the most efficient health systems in the region, whereas Venezuela and Guatemala are the least efficient.

